# The *De Novo* Transcriptome and Its Functional Annotation in the Seed Beetle *Callosobruchus maculatus*

**DOI:** 10.1371/journal.pone.0158565

**Published:** 2016-07-21

**Authors:** Ahmed Sayadi, Elina Immonen, Helen Bayram, Göran Arnqvist

**Affiliations:** Animal Ecology, Department of Ecology and Genetics, Uppsala University, Uppsala, Sweden; University of Basilicata, ITALY

## Abstract

Despite their unparalleled biodiversity, the genomic resources available for beetles (Coleoptera) remain relatively scarce. We present an integrative and high quality annotated transcriptome of the beetle *Callosobruchus maculatus*, an important and cosmopolitan agricultural pest as well as an emerging model species in ecology and evolutionary biology. Using Illumina sequencing technology, we sequenced 492 million read pairs generated from 51 samples of different developmental stages (larvae, pupae and adults) of *C*. *maculatus*. Reads were *de novo* assembled using the Trinity software, into a single combined assembly as well as into three separate assemblies based on data from the different developmental stages. The combined assembly generated 218,192 transcripts and 145,883 putative genes. Putative genes were annotated with the Blast2GO software and the Trinotate pipeline. In total, 33,216 putative genes were successfully annotated using Blastx against the Nr (non-redundant) database and 13,382 were assigned to 34,100 Gene Ontology (GO) terms. We classified 5,475 putative genes into Clusters of Orthologous Groups (COG) and 116 metabolic pathways maps were predicted based on the annotation. Our analyses suggested that the transcriptional specificity increases with ontogeny. For example, out of 33,216 annotated putative genes, 51 were only expressed in larvae, 63 only in pupae and 171 only in adults. Our study illustrates the importance of including samples from several developmental stages when the aim is to provide an integrative and high quality annotated transcriptome. Our results will represent an invaluable resource for those working with the ecology, evolution and pest control of *C*. *maculatus*, as well for comparative studies of the transcriptomics and genomics of beetles more generally.

## Introduction

Beetles (Coleoptera) are by far the most species rich taxonomic order on our planet, containing some 25% of all known animal species, and they show a tremendous range of adaptations to different food resources and habitats [1]. Beetles are generally keystone species in terrestrial ecosystems and many are serious pests in agriculture and forestry and are thus of tremendous economic importance. Yet, only a few beetle genomes have been sequenced so far [2,3], and the genomic resources available to study molecular evolution in beetles are very limited relative to many other groups. Efforts to improve this situation are complicated by the fact that many beetle genomes are fairly large (average C value = 0.7; range 0.2–5.0) and show a high repeat content [4]. Here, transcriptome assembly provides a cost effective remedy [5].

The seed beetle *Callosobruchus maculatus* Fabr. (Coleoptera: Bruchidae), also known as the cowpea beetle, is a major cosmopolitan pest of a variety of legume crops. It causes an estimated annual crop loss of > 30 million US dollars in Nigeria alone [6]. It is also an emerging model system in several areas of evolutionary biology [7–10]. Seed beetles have a very rapid life cycle (about three weeks). Females lay their eggs on the external surface of seeds. After a few days, the larvae hatch and burrow inside the seed. They feed and pupate inside the seed, before emerging as adults. Adults requires neither water nor food to reproduce successfully [11].

The genome of *C*. *maculatus* is fairly large (1.2 Gb) and shows a very high repeat content [12] and we thus decided to assemble its transcriptome. Over the past few years, there has been a marked improvement in sequence technologies (increasing the sequence reading length) and in *de novo* transcriptome assembly software tools (assembling transcriptomes without a reference genome). This approach has recently been used to assemble a few beetles transcriptomes *de novo* [13–15]. In the current transcriptome assembly effort, we sequenced samples from *C*. *maculatus* larvae, pupae and adults using an Illumina paired-end sequencing platform. Sequences were then assembled using Trinity, a *de novo* assembly software [5]. More than 145 thousand genes were generated and annotated using the Trinotate pipeline (https://trinotate.github.io/) and Blast2GO [16]. Our aim was to provide the first inclusive annotated transcriptome of *C*. *maculatus*. We also assess the degree to which the transcriptome is shared across developmental stages and ask how the size of the transcriptome changes over ontogeny, which is important both for studies of differential expression and comparative purposes. Our results will provide an important resource for gene discovery and comparative genomics of beetles and for future applied and fundamental scientific studies of the seed beetle *C*. *maculatus*.

## Materials and Methods

### Samples

*Callosobruchus maculatus* from the South India SI4 reference population were used here. This is an isogenic stock produced by 5 consecutive generations of full-sib mating to reduce the level of heterozygosity. They were reared on mung beans in laboratory climate cabinets at 29°C, 60% RH and a 12 L: 12 D light cycle. RNA material was extracted and prepared from beetles of several developmental and physiological stages to increase the diversity of expressed transcripts, namely larvae, pupae and mated and virgin adults of both sexes. In total, we prepared 11 different types of samples. A larval sample was prepared by pooling 6 individuals of different larval instars. A pupal sample was prepared by pooling 2 pupal individuals. An adult mix sample was prepared by pooling 4 adult individuals, 2 males and 2 females, randomly chosen from a population containing mated young and old beetles, fed with 5% glucose-water and dried yeast supplement. For the other 8 sample types, adult beetles were collected immediately after emergence from beans and were isolated individually without access to food. Pairs were allowed to mate, after which the sexes were separated and kept with beans for 24h. The virgins were treated in the same precise manner, but were not mated. From these beetles, we created samples representing, in an orthogonal manner, males vs. females, mated vs. virgins and abdomen vs. head/ thorax (i.e., 2 ×2 ×2 = 8 sample types, each replicated three times). All beetles were snap-frozen with liquid nitrogen. For the adult samples, the abdomen was separated from the head and thorax on ice, making sure that the tissues did not thaw. Each sample for RNA extraction was prepared by pooling body parts from 6 individuals.

### RNA extraction and purification

RNA was extracted using RNAeasy Mini Kit (Qiagen), following the manufacturer's protocol. DNase digestion was applied using DNase I (RNase-Free DNase set by Qiagen). The RNA quality and quantity was assessed and affirmed using NanoDrop, Qubit and Bioanalyzer.

### cDNA library generation and Illumina sequencing

In total, 51 sample libraries were allocated to three lanes (labelled L5, L6 and L7). Three samples were sequenced on lane 5: (1) pupae, (2) entire larvae and (3) the mix of adult males and females. The rest of the adult samples were sequenced on lanes 6 and 7, which represent two technical replicates of each of the three biological replicates of the 8 different types of samples abbreviated as: (1) AMf: Abdomen mated female, (2) AMm: Abdomen mated male, (3) AVf: Abdomen Virgin female, (4) AVm: Abdomen Virgin male, (5) HtMf: Head and thorax mated female, (6) HtMm: Head and thorax mated male, (7) HtVf: Head and thorax virgin female, (8) HtVm: Head and thorax virgin male (i.e., 3 + 2 × 8 × 3 = 51 samples in total; see S1 Table).

The RNA-seq libraries were prepared from 1μg total RNA using the Illumina TruSeq stranded mRNA sample preparation kit. At a first step, Poly-A RNA was purified from total RNA using poly-T oligo attached magnetic beads. After the purification step, mRNA is fragmented into small pieces. Fragmented RNA is then reverse transcribed to first strand cDNA using random primers. A second strand cDNA synthesis step with the incorporation of dUTP instead of dTTP is realized to achieve strand specificity. cDNA fragments are then ligated to adapters. At the final step, cDNA are purified and enriched with PCR to create a cDNA library. All sequencing was performed using Illumina HiSeq 2500 sequencing technology with a maximum read length of 2x100 bp. The paired-end library was prepared using the TruSeq stranded mRNA Sample Preparation kit according to the manufacturer’s guidelines [17]. The library generation and sequencing were performed by the SNP&SEQ Technology Platform at Uppsala University.

### Bioinformatic analyses

#### Quality trimming

RNA reads obtained from sequencing where quality assessed using FastQC v.0.11.2 [18]. Illumina adapter sequences left in reads were removed using cutadapt v.1.2.1 [19]. Cutadapt will search for a supplied list of adapters in all the reads, a minimum overlap of 15bp between the adapter and the read is required. The adaptor search is done twice in each read to remove adaptors in tandem. Low quality reads towards the 3’ and 5’ ends of the reads were trimmed with Trimmomatic v.0.3; reads were scanned with a 4 base wide sliding window, and leading or trailing bases with average phred quality score lower than 20 were dropped. Reads with a length lower than 50bp were also discarded [20].

#### Transcriptome de novo assembly

Digital normalization and transcriptome *de novo* assembly was conducted using the Trinity 2.0.6 software with a default k-mer size of 25. Trinity is composed of three different modules: Inchworm, Chrysalis and Butterfly. Inchworm builds a K-mer dictionary from the reads, which will lead to the construction of contigs. Chrysalis connects all overlapped contigs into components using a de Bruijn graph approach. In a final step, Butterfly simplifies all the generated graphs to report full-length transcripts and their alternatively spliced form [21]. DeconSeq standalone version 0.4.3 [22] was used to detect and remove sequence contaminations from the assembled transcriptome, using bacterial, fungal, plant, virus and other databases. DeconSeq was run with alignment identity threshold of 95% (-i 95) and alignment coverage threshold of 90% (-c 90). CD-HIT-EST version 4.6.1 (2012-08-27) was subsequently used for clustering of assembled transcripts with the default parameters at two different sequence identity thresholds (100% and 98%).

In order to statically assess the quality of the assembled transcriptomes, we assessed the number of paired-end reads that were present in the assembled transcripts. To achieve this, we used Bowtie (version 0.12.6) [23] to align all raw reads back to the assemblies. In order to avoid an overestimation of transcriptome quality during mapping, only one position was reported for reads that mapped to several locations.

#### Transcriptome function annotation

**Fig 1:**
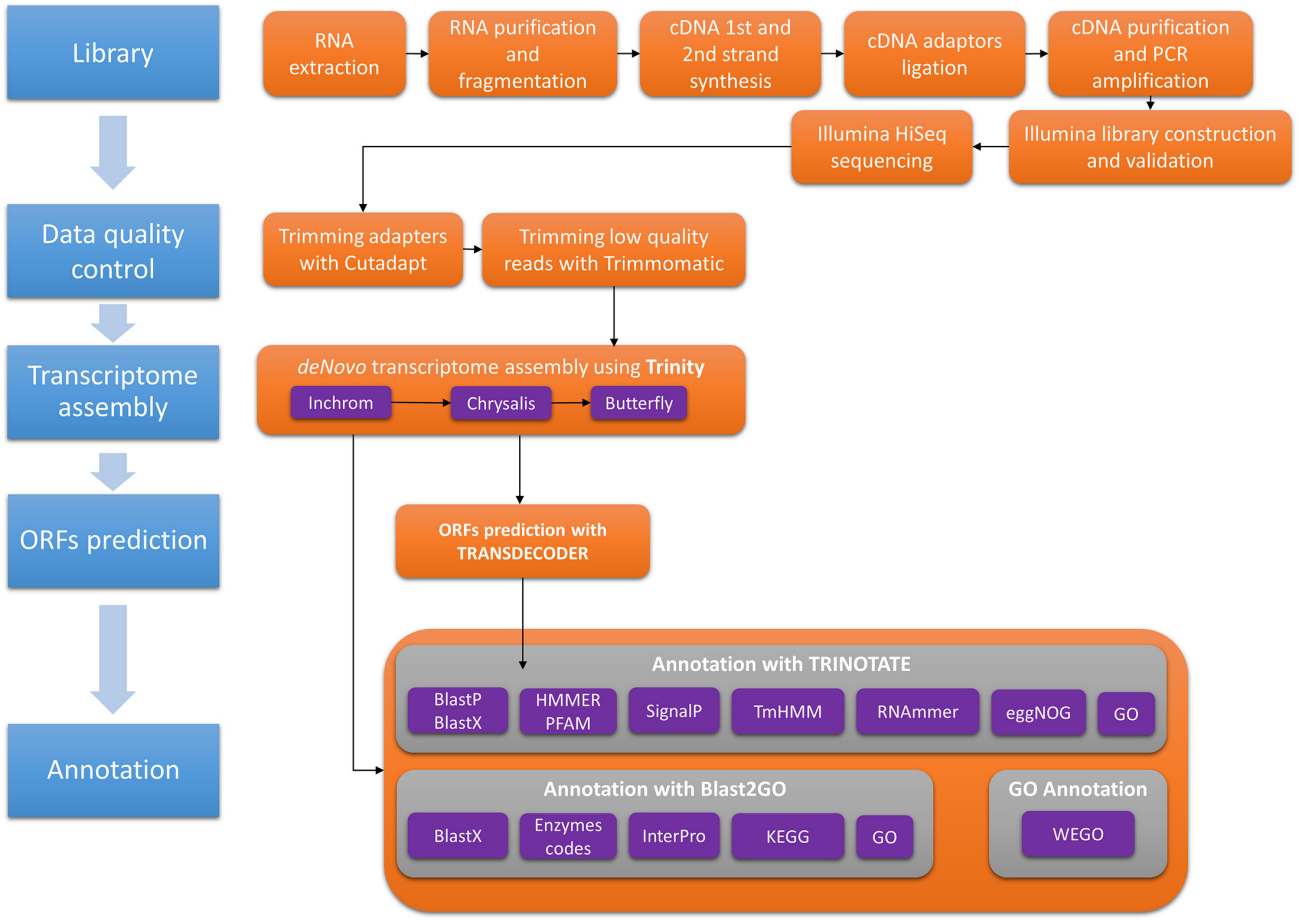
The overall workflow, summarizing the steps of the transcriptome assembly.

Annotation was performed using Blast2go version 3.2 [16] and the Trinotate pipeline (https://trinotate.github.io/). All assembled putative genes (henceforth, genes, for brevity) were searched against several databases (the NCBI (non-redundant) protein database (Nr) (ftp://ftp.ncbi.nih.gov/blast/db/ 29-02-2015), Swissprot-Uniprot database, Kyoto Encyclopedia of Genes and Genomes (KEGG), GO (Gene Ontology), EggNog and InterproScan) using BlastX with an E-value cut-off set to 10^−5^ [24,25]. Gene open reading frames (ORFs) were predicted using Transdecoder v.2.0.1 (http://transdecoder.sourceforge.net/). We retained only predicted ORFs that were at least 100 amino acids long, whether partial or complete. Obtained ORFs where blasted using BlastP against the NCBI Uniref90 database with an E-value cut-off of 10^−6^ [24]. The remaining functional annotation was achieved using Blast2GO and Trinotate. The Trinotate pipeline uses several software: Hmmer v.3.1b1, a protein domain identification (PFAM) software [26], Tmhmm v.2.0c prediction of transmembrane helices in proteins [27], Rnammer v.1.2 to predict ribosomal RNA [26], SignalP v.4.1 predict signal peptide cleavage sites [28,29], prediction of gene ontology GOseq [30], eggnog v.3.0 search for orthologous group [31]. The gene completeness of the assembled transcriptome was assessed using the BUSCO (Benchmarking Universal Single-Copy Orthologs) library (http://busco.ezlab.org/) [32]. Blast2GO uses the KEGG database and InterProScan software [33]. The overall workflow, summarizing the transcriptome assembly steps, is presented graphically in Fig 1.

### Availability of supporting data

Raw RNA-Seq data is deposited in FASTQ format to the NCBI Sequence Read Archive database (SRA) under the BioProject accession number PRJNA309272. The three separate as well as the combined assembly have been deposited at DDBJ/EMBL/GenBank under the accession numbers GEUD00000000, GEUH00000000, GEUE00000000 and GEUF00000000. The versions described in this paper are the first versions (GEUD01000000, GEUH01000000, GEUE01000000 and GEUF01000000).

## Results and Discussion

### Experiment design

For the transcriptome assembly and analysis, RNA material was extracted and prepared from beetles in different ontogenetic stages (larvae, pupae or adults), from both sexes in different reproductive conditions (virgin or mated), using different body parts (head and thorax, abdomen or whole body). In total, 27 different samples (libraries) of 11 different types were sequenced (for details, see material and methods). For adults, technical and biological replicate samples were sequenced separately to allow assessment of sex-specific differential expression, which will be analyzed and reported in a separate study. The RNA-seq libraries were generated using the Illumina TruSeq stranded mRNA sample preparation kit, generating 101 base paired-end reads [34].

### Sequencing and *de novo* assembly

In total, 492 million pairs of 101 base length paired-end reads were generated using Illumina HiSeq sequencing platform. Table 1 summarizes sample statistics for all reads.

**Table 1:** Summary statistics of sequencing data and the combined *de novo* transcriptome assembly of *C*. *maculatus*.

**Read processing**	
Raw reads (2×101 bp)	492,095,358
Filtered Paired-end reads (2×101 bp)	474,915,945
**Trinity *de novo* Assembly**	
Total assembled bases	199,346,342
Number of Transcripts	218,192
Number of genes	1465,883
Average transcript length	914
Min gene length	224
Max gene length	26,805
Number of genes > 1 Kb	26,215
Number of genes > 5 Kb	1,443
Number of genes > 10 Kb	107
Transcript N50 (bp)	1,818
GC content	38.98

All sample reads were combined to generate a single combined reference transcriptome assembly. Before assembling reads into transcripts, however, raw reads were quality filtered. To that aim, we first assessed read quality using the FastQC software. This showed a high Phred quality score (average score over all sequences > 38 Phred) but indicated a small bias in the first 10 bases in reads. To check if this potential bias was due to adaptor sequences being left from the sequencing step or to low quality bases, we applied the Cutadapt and Trimmomatic softwares to clean the reads. As a result, Cutadapt trimmed 42 adaptors sequences from several reads and Trimmomatic removed low quality bases and dropped reads with length lower than 50 bases. In our case, single-end reads were few and we therefore only used paired-end reads for the transcriptome assembly. In the end, a total of 474,9 million pairs of reads (96.6%) where retained and used for the assembly [35].

Due to the high number of reads, a digital normalization step was needed prior to the assembly, to down-scale reads occurring at high coverage and discard reads with an aberrant k-mer abundance profile. This step reduces the number of reads, but increases the quality of the assembly by removing non-uniform and redundant k-mer reads, thus improving the assembly process time. All combined filtered reads were thus digitally normalized and *de novo* assembled with a default k-mer of 25, using the Trinity assembler [36]. Trinity assembled transcripts were then decontaminated by discarding contaminant sequences (e.g. bacterial, fungal, plant) from the transcriptome, using a combined approach: we first ran DeconSeq to remove contaminant transcripts and then relied on annotation to remove additional contaminant transcripts (for details, see “Annotation and functional assignment”). A total of 9632 (4.2%) transcripts were considered as probable contaminant and discarded. Trinity generated 218,192 contamination-free transcripts which corresponded to 145,883 genes, with a N50 length of 1,818 bases and a mean transcript size of 914 bases. Transcript length ranged from 224 bases to 26,805 bases, with 54,358 transcripts being >1 kb and 283 being >10 kb. These primary assembly statistics imply that transcripts were well assembled and could potentially code for full-length proteins sequences. GC content of the final assembly was 39%, which is close to the GC content of the raw reads (41–43%). An in-house Perl script was used to extract the longest transcript for each gene, which were considered for the downstream analysis as representatives for each cluster of transcripts. A detailed summary of the assembly statistics is provided in Table 1.

It is well known that *de novo* transcriptome assemblies produce many more transcripts, especially at high coverage, than a normal annotation [37,38]. *De novo* transcriptome assemblies are faced with a number of issues, such as coverage variation between highly and low expressed transcripts, polymorphism, alternative splicing, chimeric transcripts and identical sequences repeated in different genes, making the reconstructing of full-length transcripts without redundancy computationally challenging. One possibility is to assess the level of redundancy in a given transcriptome assembly is to perform a cluster analysis based on sequence similarity. In our case, however, clustering (using CD-HIT-EST) reduced the number of transcripts marginally: by 1% and 20% with a sequence identity threshold set to 100% and 98%, respectively. Considering the fact that clustering did not much reduce the number of transcripts, in combination with the fact that clustering risk collapsing valid isoforms, paralogs, and may introduce chimeras, we decided to not rely on clustering but to retain all transcripts [39,40].

Our analysis showed that 84% of the 145,883 genes are represented by one isoform, 7% of the genes are expressed with two isoforms and 9% of the genes are expressed with three or more isoforms. Fifty isoforms was the maximum number of isoforms for a single gene. The high number of genes and isoforms may be due to the fact that the *C*. *maculatus* transcriptome was generated by combining reads originating from different development stages. To test this, we generated (in parallel with the main combined transcriptome assembly) separate assemblies for the different development stages (e.g. larvae, pupae or adults) (Table 2). These more homogenous assemblies showed higher N50 values and mean transcript length, but showed a much lower number of transcripts and genes compared to the combined assembly. We also note that the higher number of isoforms per gene in the combined assembly suggest that a number of genes are expressed with different isoforms in different samples (i.e., in different ontogenetic stages). Finally, the raw paired-end reads of each sample were mapped back to the assembled transcriptome to assess read content, because fragmented or short transcripts may only align to one fragment read of a pair. We found that more than 82% of the reads were correctly mapped as proper pairs (S1 Table). This shows that most genes were properly assembled.

**Table 2:** Summary statistics of the individual and the combined transcriptome assemblies.

	Larvae	Pupae	Adults	Combined
**Transcripts**	72,299	79,647	71,523	218,192
**Genes**	57,061	62,374	53,793	145,883
**N50**	1,819	1,969	2,072	1,818
**Mean contig length**	953	962	1,037	914
**GC content**	39.52	39.33	39.34	38.98
**Total assembled bases**	68,882,917	76,609,446	74,156,506	199,346,342
**Transcripts > 1 kb**	19,219	21,366	21,617	54,358
**Transcripts > 5 kb**	1,254	1,606	1,576	3,889
**Transcripts > 10 kb**	93	130	132	283

### Assessment of completeness

As a complementary approach to assess the quality of the *C*. *maculatus* transcriptome, besides statistics such as the N50 value and the number of genes longer than 1kb, we assessed transcriptome completeness in terms of gene content. We searched the transcriptome for the presence or absence of a list of conserved orthologous genes. We used the BUSCO (Benchmarking Universal Single-Copy Orthologs) library of Metazoa orthologous genes [32]. This represents a collection of 843 single-copy metazoan orthologs, well-annotated and conserved.

We obtained 760 (90%) complete BUSCO hits, and duplicate hits to 428 (50%) genes. We found that another 27 (3.2%) were fragmented and 56 (6.5%) were missing. The relatively high number of duplicates may, in theory, represent allelic variation (heterozygosity) in the sample used to construct the assembly, gene duplication and/or mechanisms such as alternative splicing. The fact that heterozygosity is very low indeed in our stock population suggest that allelic variation should contribute little. In an attempt to better understand the origin of our duplicates, we ran the same BUSCO analysis using the three individual assemblies. This analysis still showed a high number of completely recovered genes (87% for larvae, 88% for pupae and 89% for adults), but a markedly lower number of duplicates (26%, 28% and 25% respectively). This suggests that gene duplication and/or alternative splicing, with stage-specific expression of paralogs and/or isoforms, may contribute importantly. In any case, the high number of complete and duplicate genes that was recovered provides an important validation of the depth and completeness of the assembly.

### Annotation and functional assignment

The annotation is arguably the most important part of our analysis, as it enables us to evaluate and interpret the content of the *C*. *maculatus* transcriptome assembly. We initiated the annotation by blasting the transcriptome, using BLASTx, against the Nr (ncbi non-redundant) database with a cut-off E-value set to 10^−5^. In total, 37,990 (26%) genes showed a significant hit in the Nr database. Almost 90% of the blast hits belonged to Metazoan taxa, the rest representing hits with viruses, bacteria, fungi, and viridiplantae [41]. These genes were considered as likely contaminants and were dropped. In total, we retained 33,216 genes with significant blast hits in our downstream analysis.

**Fig 2:**
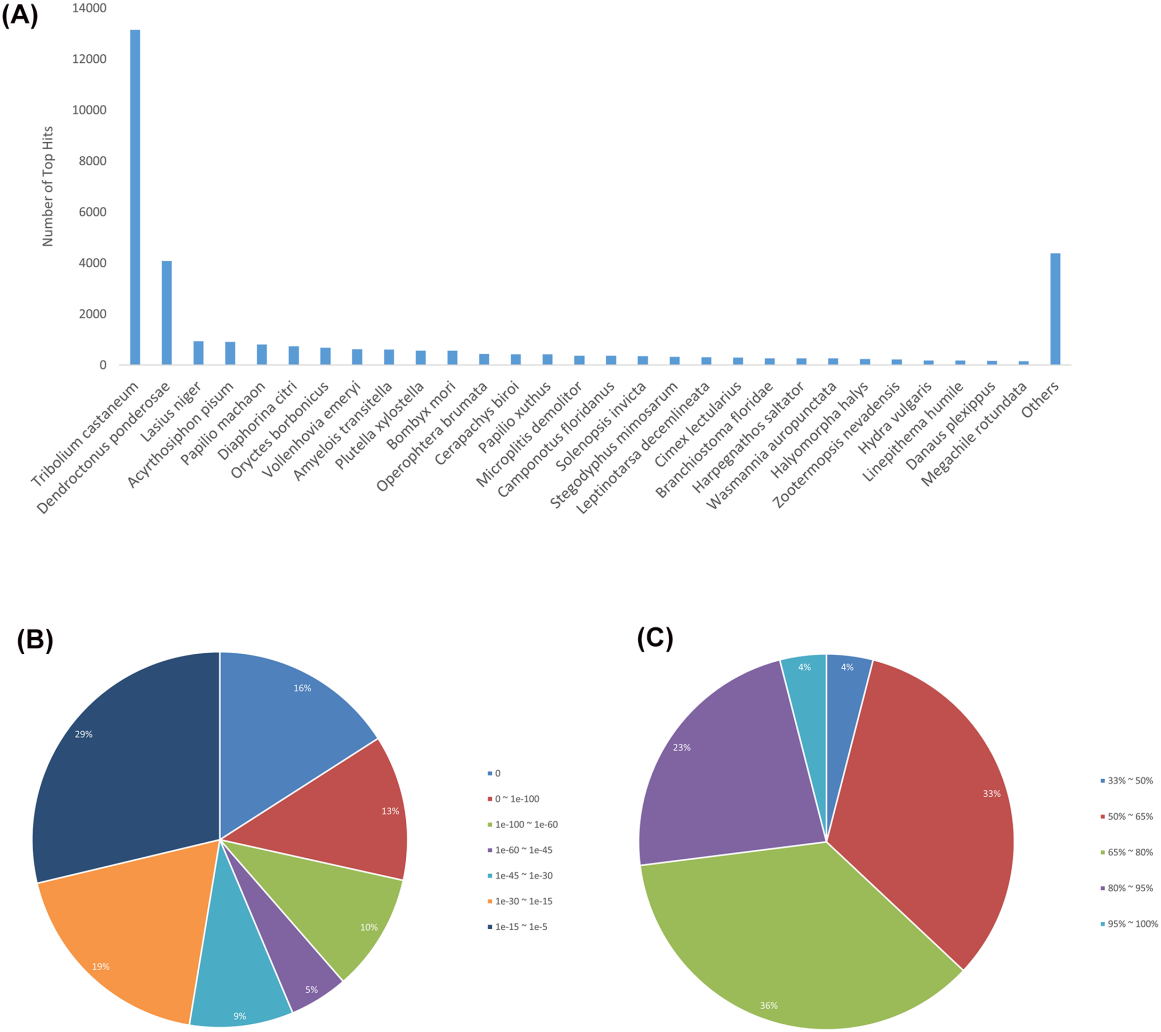
Blast2GO blast results. **(A)** Species distribution for the top BLAST hits for genes in the Nr database. **(B)** E-value distribution of BLAST hits with a cutoff E-value of 1.0E-5. **(C)** Similarity distribution of the top BLAST hits.

The blast hits distribution in the Nr database showed most hits with *Tribolium castaneum* (Coleoptera, Tenebrionidae). More than 40% of the genes showed a similar sequence in *T*. *castaneum*. The second top blast hit taxa was *Dendroctonus ponderosae* (Coleoptera, Curculionidae), with 12% similar genes. This is reassuring, considering that these three beetle species belong to the same infraorder (Cucujiformia). The remaining blast hits showed similarities to other insects in the majority of cases (Fig 2A). The E-value and sequence similarity distribution of the top blast hits for each gene add strength to the blast analysis and to the general quality of the assembled genes; more than 16% of the genes have a blast hit E-values equal to zero (Fig 2B), and more than 59% of genes showed sequence identity higher than 65% with the best Nr database hit (Fig 2C).

We next evaluated the ability of the predicted genes to reconstruct full-length proteins. We scanned all gene sequences for open reading frames (ORFs), using Transdecoder [21]. We obtained 28,744 genes (17,455 with complete ORFs) with ORFs longer than 100 amino acids, with the longest ORF being 8,593 amino acids and the average ORF length being 404 amino acids. No less than 87% of the genes with ORFs show blast hits in the Nr database and these have an average length of 448 amino acids. The large number, and high sequence length, of the predicted ORFs provided a further validation of the quality of the assembly.

Further functional annotation and GO term assignments was conducted using the Blast2Go software and the Trinotate pipeline [16,21]. The Trinotate pipeline incorporates several annotation methods: Blast homology search against SwissProt and Uniref90 databases, Pfam domain prediction, protein signal peptide (SignalP), transmembrane domain (tmHMM) prediction, and comparison to the EggNog database of orthologous group, which also includes the COG database [31]. Blast2GO apply a similar approach, where best Nr (NCBI non-redundant) database blast hits are used to map GO terms, retrieve KEGG pathways and to scan InterProScan signatures for protein domains detection. Blast2GO and Trinotate annotation files are supplied in S1 and S2 Files.

### Gene Ontology

Gene ontology (GO) (http://geneontology.org/) is an internationally standardized functional classification system for genes, aimed to describe the properties of genes and their product within an organism using a dynamic-updated controlled vocabulary. GO comprise three main categories: molecular function, cellular component and biological process [42]. Blast2GO mapping was used to obtain the GO annotation based on the gene blast hits from the Nr database, and then completed using Inteproscan and ANNEX annotation. All GO terms were then functionally classified using the WEGO (Web Gene Ontology Annotation Plot) software [43].

**Fig 3:**
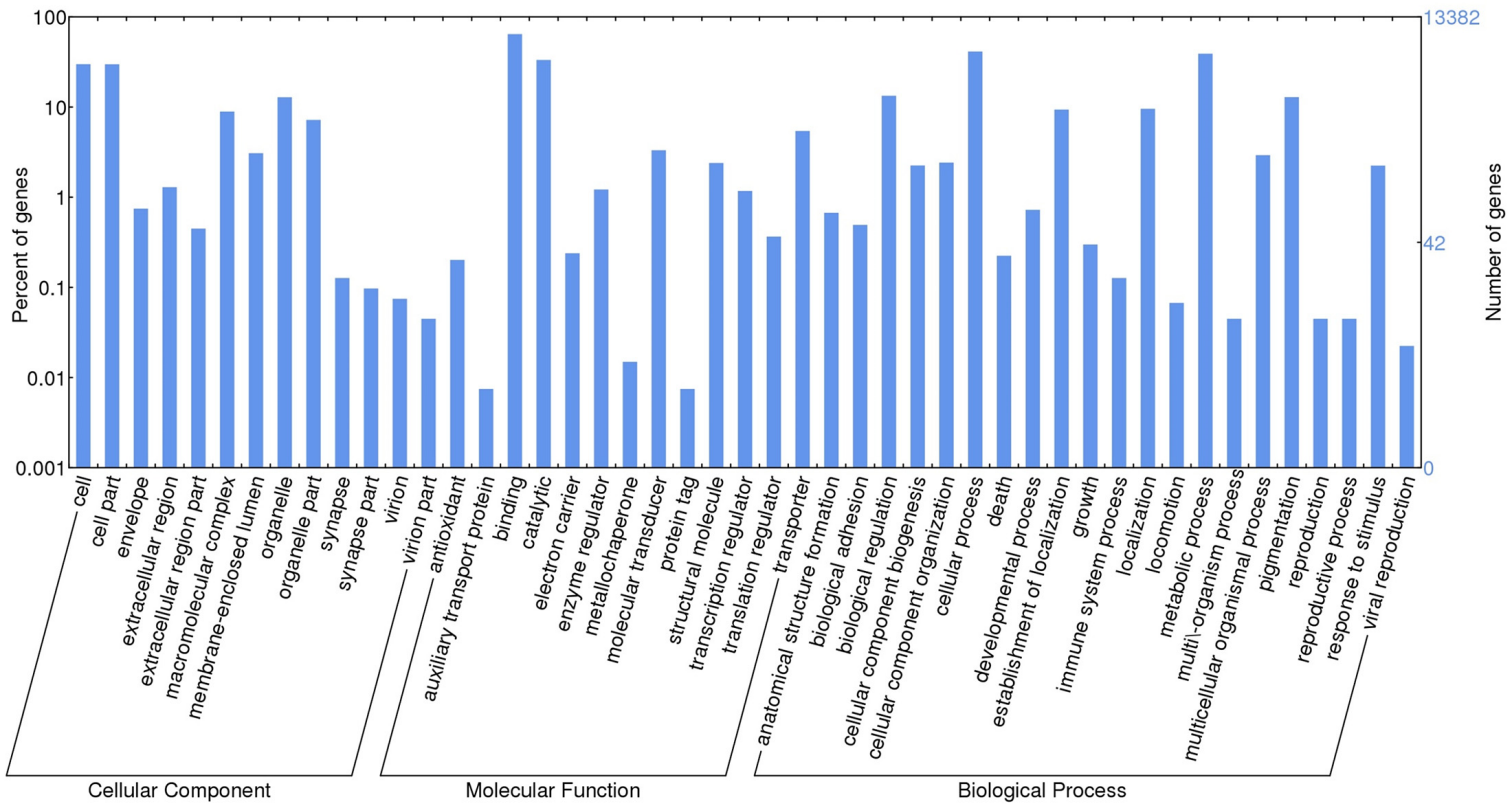
Histogram of GO classifications of *C*. *maculatus* Unigenes.

In total, 34,100 GO terms where assigned to 13,382 genes (45% of the genes with Nr blast hits). The majority of the GO terms were assigned to molecular function (17,911, 52%), followed by biological process (11,108, 33%), and cellular compartment (5,081, 15%). The terms were derived from 47 different functional groups (GO sub-categories level 2) (Fig 3). Within molecular function, the largest proportion was assigned to binding (64.5%), and catalytic activity (33.3%) categories; within biological process: cellular process (41.3%), metabolic process (39.1%), biological regulation (13.3%), pigmentation (12.9%), and localization (9.6%), were over-represented; and within cellular compartment, the majority were assigned to cell (29.9%), cell part (29.8%) and organelle (12.8%) categories. An over-representation of these categories has also been seen in the transcriptome annotations of other beetles [44,45].

Almost all of the GO terms were inferred from electronic annotation (IEA), using gene annotation that had blast matches to proteins belonging to the UniprotKB database [46]. UniprotKB is a well-annotated database, composed by manually and automatically annotated records sections. These results imply that GO annotation is mainly driven by blast matches found in the UniprotKB databases. Similar results were obtained for GO annotation using Trinotate pipeline.

### COG classification

**Fig 4:**
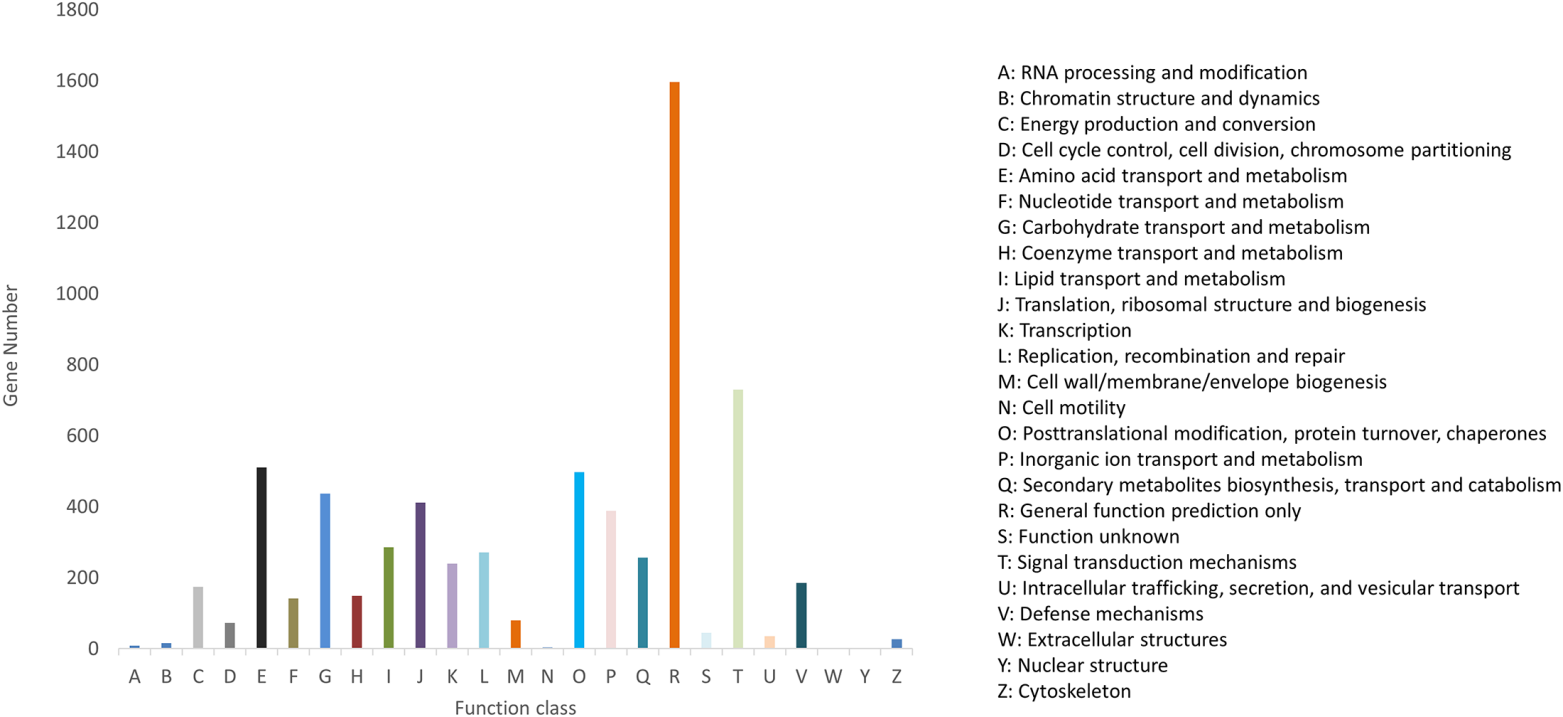
Histogram of the clusters of orthologous groups (COG).

The Clusters of Orthologous Groups (COG) [47], is a database where orthologous gene products are classified into 25 functional categories. It is based on the principle that conserved genes should be classified according to their homologous relationship. Each COG consists of individual orthologous proteins, typically sharing the same general function. COG screening was performed using the EggNog database [31], integrated within the Trinotate pipeline. All genes where aligned to the COG database to predict and classify their functions. In total 5,475 (13% of the genes with Nr blast hits) genes (14,060 transcripts) were assigned to 25 COG functional categories (Fig 4). The largest group is represented by the cluster for general function prediction (1,596, 29%), followed by signal transduction mechanisms (729, 13%), amino acid transport and metabolism (510, 9%), posttranslational modification, protein turnover, chaperones (497, 9%), carbohydrate transport and metabolism (437, 8%), and translation, ribosomal structure and biogenesis (411, 7%). A few other clusters, such us chromatin structures and dynamics, RNA processing and modifications, cell motility, extracellular structures, and nuclear structure, are underrepresented or absent. Similar gene COG classifications distributions has been found in other beetles (e.g. pine sawyer beetle, pine shoot beetle) [44,48].

### KEGG pathway analysis

**Fig 5:**
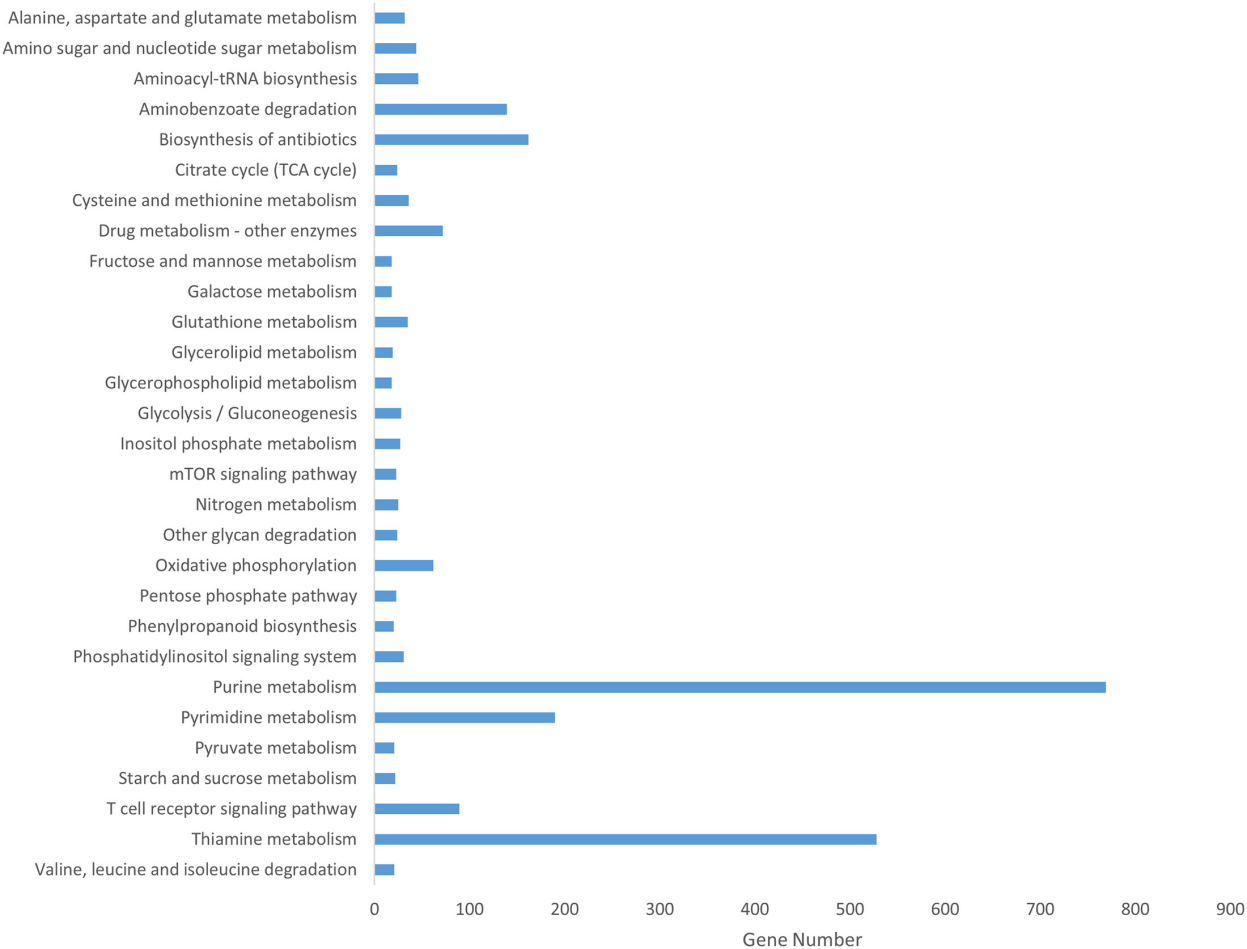
KEGG pathway distribution.

To better understand functions and interactions, all annotated genes were mapped against the KEGG database for a pathway-based analysis. As a result, a total of 3,127 genes were assigned to a KEGG pathway. This relatively low number of genes assigned to a pathway is likely the result of imperfect annotation caused by Blast2go, although genes were present in 116 different KEGG pathways. KEGG pathways distribution is summarized in Fig 5. The top 5 pathways are purine metabolism (769, 24.6%), thiamine metabolism (528, 16.9%), pyrimidine metabolism (190, 6%), biosynthesis of antibiotics (162, 5.2%), Aminobenzoate degradation (139, 4.5%).

### Transcriptome sharing

Using our inclusive combined assembly, we quantified transcript abundance using the RSEM package through the Trinity pipeline, where all reads were re-aligned to each transcript with Bowtie (short read aligner). Relative abundance of each transcript or gene was reported as Fragments Per Kilobase per Million mapped reads (FPKM). Using this route, we identified 114,249 (78.3%) genes as being actively expressed in all of our samples (FPKM > 0.5). To assess the degree to which the transcriptional profile is shared or private across ontogentic stages (larvae, pupae and adults), we compared the presence and absence of genes between the three of them. For example, genes with a FPKM value > 2 in the larvae stage and with FPKM = 0 in pupae and adults, were considered private to the larvae stage. This comparison identified 51 annotated genes specific to larvae, 63 genes specific to pupae and 171 genes specific to adults (Table 3) (S3 File). Overall, the comparison of the three assemblies suggests that adults shows the highest, and larvae the lowest, transcriptional diversity and privacy. This is also consistent with the fact that the adult assembly contained the largest number of long transcripts (Table 2). The increase in the number of expressed genes during ontogeny illustrates the importance of including samples from several developmental stages, of which adults should be one, in order to construct inclusive *de novo* assemblies. In Table 4, we summarized a selection of the top blast hit genes specific to larvae, pupae, and adults.

**Table 3:** The number of private genes during ontogeny in *C*. *maculatus*.

		All	ORFs	Blast Nr	ORFs with BlastNr
**Genes**		145,883	27,878	33,129	22,401
	FPKM> = 2	212	51	51	38
**Larvae**	2>FPKM> = 0.5	1,623	114	179	80
	2>FPKM>0	8,453	895	1,288	628
	FPKM> = 2	531	64	63	35
**Pupae**	2>FPKM> = 0.5	2,946	159	266	101
	2>FPKM>0	14,823	1,437	2,197	1,017
	FPKM> = 2	455	222	171	151
**Adults**	2>FPKM> = 0.5	2,365	367	447	283
	2>FPKM>0	16,669	2,196	2,894	1,593

Here, private genes are defined as those expressed at low (either 2>FPKM >0 or 2>FPKM> = 0.5) or higher levels (FPKM> = 2) in a particular developmental stage, but not found expressed in any of the other stages (FPKM = 0).

**Table 4:** The top expressed private genes in larvae, pupae and adults.

	Genes id	Predicted Function (Blast2GO)	Length	FPKM
	TR64718|c0_g1_i3	larval cuticle protein	684	2.802
	TR24068|c0_g1_i4	catalase-like	1619	7.605
	TR28212|c0_g1_i1	equilibrative nucleoside transporter 3- partial	262	2.85
**Larvae**	TR8452|c1_g2_i3	glyoxylate reductase hydroxypyruvate reductase-like	1740	15.646
	TR1265|c0_g1_i1	glycosyl hydrolase	1335	22.887
	TR52474|c1_g4_i1	glycoside hydrolase family 1	579	149.367
	TR18717|c0_g2_i1	beta-galactosidase-1-like protein 2	2098	3.517
	TR55315|c3_g1_i2	cathepsin b-like cysteine protease	1515	17.053
	TR55185|c0_g2_i1	cuticle protein 7	1477	3.359
	TR68734|c1_g2_i1	resilin isoform x1	2028	164.682
**Pupae**	TR73641|c7_g7_i1	cuticle protein 8-like	807	52.115
	TR7965|c0_g2_i1	endothelin-converting enzyme 2-like	456	2.292
	TR10464|c0_g1_i1	myosin-VIIa	3706	5.021
	TR16797|c0_g1_i1	probable h aca ribonucleoprotein complex subunit 1	1510	12.089
	TR64463|c0_g1_i1	tektin-2	1565	6.473
	TR20413|c0_g1_i1	tubulin alpha-1 chain	1667	7.276
**Adults**	TR9448|c0_g1_i2	odorant-binding protein 4	542	5.003
	TR29765|c3_g1_i1	bone morphogenetic protein 10 isoform x2	1287	2.578
	TR2044|c0_g1_i1	calmodulin isoform x1	899	2.811
	TR37403|c3_g1_i11	digestive cysteine protease intestain	2269	2.088

#### Larval genes

Digestive enzymes dominated here. Cathepsin b-like cysteine protease 2 is one of the genes specific to the larval stage. This gene is a critical component of digestive pathways in larvae, and was also found highly expressed in the larval stage of *Tribolium castaneum* [49]. Other highly expressed genes private to larvae were Glycoside Hydrolase family 1 (FPKM 149.37) and Glycosyl Hydrolase (FPKM 22.89). The Glycoside Hydrolase family comprises other known enzymes, which were also present in larvae such us the Beta-galactosidase-1-like protein 2 (FPKM 3.52). These genes are generally present in gut tissues and are implicated in the chitin degradation process [50]. We also found a larval cuticle protein expressed only in larvae, which is an important gene for the development of the cuticle of the larval body wall [51].

#### Pupal genes

In pupae, we found two private cuticle class genes; Cuticle protein 8-like (FPKM 52.12) and Cuticle protein 7 (FPKM 3.36). These genes are important for the formation and development of insect cuticle [52], a key process during the mid- and late stages of pupation. Other private pupal genes are the Resilin isoform x1 (FPKM 164.68) and myosin-VIIa (FPKM 5). Resilin is an elastomeric protein and a crucial component for wing movement in insects [53] and Myosin is a ATP-dependent motor protein that plays a fundamental role in muscle contraction [54].

#### Adults genes

The adult stage showed private expression of the Odorant-binding protein 4 gene, which is involved in adult olfaction. Although olfaction is important for most of insects [55], odorant binding proteins are known to be important for both host- and mate-finding in adult seed beetles. Further, Tektin-2 and Tubulin alpha-1 chain were not expressed in larvae and pupae but were expressed in adults. Tektin is an essential protein for the development of cilia and flagella. Both Tektin and Tubulin are important components of the cytoskeleton doublet microtubule in insect [56], suggestion that they are important during gamete production in adults. We also found a private bone morphogenetic protein, a member of a group of proteins known to be involved in neural signaling in *Drosophila* [57].

## Conclusions

In this study, we provide a comprehensive assembly of the *C*. *maculatus* transcriptome, based on deep Illumina sequencing of diverse samples. The transcriptome covers a large number of genes expressed in all developmental stages. In total, 492 million paired-end reads were assembled into a high number of genes (145,883), of which 33,216 were annotated. We found that including samples from several developmental stages was crucial in order to provide a maximally integrative transcriptome. We believe that this data will provide a valuable resource for future studies of the seed beetle *C*. *maculatus* as well as for comparative gene expression and genomic analyses of beetles more generally.

## Supporting Information

S1 File Transcriptome annotations with Blast2GO. (ZIP)

S2 File Transcriptome annotations with Trinotate. (ZIP)

S3 File Expressed private genes in larvae, pupae and adults, annotated with Blast2GO. (ZIP)

S1 Table Summary statistics of the RNA-Seq data. (DOCX)
