# Supplementary material for: The De Novo Transcriptome and Its Functional Annotation in the Seed Beetle Callosobruchus maculatus
Source: PLoS One. 2016 Jul 21;11(7):e0158565. doi: 10.1371/journal.pone.0158565 (PMC4956038; doi:10.1371/journal.pone.0158565)
Supplement: S1 Table — (DOCX) [file pone.0158565.s004.docx]

**Supplementary Table 1.** Summary statistics of the RNA-Seq data.

AMf: Abdomen mated female, AMm: Abdomen mated male, AVf: Abdomen virgin female, AVm: Abdomen virgin male, HtMf: Head and thorax mated female, HtMm: Head and thorax mated male, HtVf: Head and thorax virgin female, HtVm: head and thorax virgin male. Biological replicates: biological r. Technical replicate: T. r.

|  |  | Biological r. 1 | Biological r. 2 | Biological r. 3 | All paired end reads combined | Total aligned reads | Proper  pairs | Proper  Pairs % |
| --- | --- | --- | --- | --- | --- | --- | --- | --- |
| AMf | T. r. L6  T. r. L7 | 6906254  7249598 | 6814648  7185088 | 6475248  6628909 | 41259745 | 74035957 | 64794594 | 87.52 |
|  |  |  |  |  |  |  |  |  |
| AMm | T. r. L6  T. r. L7 | 6863762  7112715 | 6349643  6643406 | 6555023  6546782 | 40071331 | 69508946 | 58222554 | 83.76 |
|  |  |  |  |  |  |  |  |  |
| AVf | T. r. L6  T. r. L7 | 6503318  6659580 | 6528073  6642122 | 6622981  6875429 | 39831503 | 71836804 | 63860312 | 88.9 |
|  |  |  |  |  |  |  |  |  |
| AVm | T. r. L6  T. r. L7 | 7003926  6911455 | 7364874  7361914 | 6572939  6813858 | 42028966 | 74148542 | 61999938 | 83.62 |
|  |  |  |  |  |  |  |  |  |
| HtMf | T. r. L6  T. r. L7 | 6451420  6633312 | 6991243  6900952 | 7036588  6966025 | 40979540 | 73989710 | 66882200 | 90.39 |
|  |  |  |  |  |  |  |  |  |
| HtMm | T. r. L6  T. r. L7 | 6655124  6928710 | 6293852  6467946 | 6405823  6463463 | 39214918 | 71321809 | 62950272 | 88.26 |
|  |  |  |  |  |  |  |  |  |
| HtVf | T. r. L6  T. r. L7 | 6629591  6564486 | 7041094  7330251 | 5988670  6292865 | 39846957 | 72192518 | 63351418 | 87.75 |
|  |  |  |  |  |  |  |  |  |
| HtVm | T. r. L6  T. r. L7 | 6675978  6726276 | 7094038  7245756 | 6750506  6967604 | 41460158 | 74405209 | 65356758 | 87.84 |
|  |  |  |  |  |  |  |  |  |
|  |  |  |  |  |  |  |  |  |
| L |  |  |  |  | 59842548 | 107492355 | 91373762 | 85 |
|  |  |  |  |  |  |  |  |  |
| P |  |  |  |  | 56130084 | 99702578 | 84893062 | 85.15 |
|  |  |  |  |  |  |  |  |  |
| Adults |  |  |  |  | 51429608 | 92784802 | 78119332 | 84.19 |
